# The associations between dysregulation of human blood metabolites and lung cancer risk: evidence from genetic data

**DOI:** 10.1186/s12885-024-12416-1

**Published:** 2024-07-18

**Authors:** Gujie Wu, Jun Liu, Haochun Shi, Binyang Pan, Min Li, Xiaolin Wang, Yao Li, Lin Cheng, Weigang Guo, Yiwei Huang

**Affiliations:** 1 Department of Thoracic Surgery, Zhongshan Hospital, Fudan University, Shanghai, 200032 China; 2 Department of Endocrinology and Metabolism, Zhongshan Hospital, Fudan University, Shanghai, 200032 China; 3 Shanghai Medical College, Fudan University, Shanghai, 200032 China; 4 Regenerative Medicine Institute, Biomedical Sciences Building, School of Medicine, National University of Ireland (NUI), Galway, Ireland

**Keywords:** Lung cancer, Metabolomics, Mendelian randomization, Genome-wide association studies, Causality

## Abstract

**Background:**

Metabolic dysregulation is recognized as a significant hallmark of cancer progression. Although numerous studies have linked specific metabolic pathways to cancer incidence, the causal relationship between blood metabolites and lung cancer risk remains unclear.

**Methods:**

Genomic data from 29,266 lung cancer patients and 56,450 control individuals from the Transdisciplinary Research in Cancer of the Lung and the International Lung Cancer Consortium (TRICL-ILCCO) were utilized, and findings were replicated using additional data from the FinnGen consortium. The analysis focused on the associations between 486 blood metabolites and the susceptibility to overall lung cancer and its three major clinical subtypes. Various Mendelian randomization methods, including inverse-variance weighting, weighted median estimation, and MR-Egger regression, were employed to ensure the robustness of our findings.

**Results:**

A total of 19 blood metabolites were identified with significant associations with lung cancer risk. Specifically, oleate (OR per SD = 2.56, 95% CI: 1.51 to 4.36), 1-arachidonoylglyceropholine (OR = 1.79, 95% CI: 1.22 to 2.65), and arachidonate (OR = 1.67, 95% CI: 1.16 to 2.40) were associated with a higher risk of lung cancer. Conversely, 1-linoleoylglycerophosphoethanolamine (OR = 0.57, 95% CI: 0.40 to 0.82), ADpSGEGDFXAEGGGVR, a fibrinogen cleavage peptide (OR = 0.60, 95% CI: 0.47 to 0.77), and isovalerylcarnitine (OR = 0.62, 95% CI: 0.49 to 0.78) were associated with a lower risk of lung cancer. Notably, isoleucine (OR = 9.64, 95% CI: 2.55 to 36.38) was associated with a significantly higher risk of lung squamous cell cancer, while acetyl phosphate (OR = 0.11, 95% CI: 0.01 to 0.89) was associated with a significantly lower risk of small cell lung cancer.

**Conclusion:**

This study reveals the complex relationships between specific blood metabolites and lung cancer risk, highlighting their potential as biomarkers for lung cancer prevention, screening, and treatment. The findings not only deepen our understanding of the metabolic mechanisms of lung cancer but also provide new insights for future treatment strategies.

**Supplementary Information:**

The online version contains supplementary material available at 10.1186/s12885-024-12416-1.

## Introduction

Lung cancer (LC) continues to be a major global health challenge and the leading cause of cancer-related deaths worldwide, accounting for more than 1.8 million deaths each year [1]. Despite advances in medical research and clinical practices, the incidence of LC is on the rise, emphasizing the critical need for more innovative diagnostic methods and effective treatment strategies [2, 3]. Metabolomics, a critical component of systems biology, has emerged as a powerful tool for uncovering the metabolic alterations that underpin cancer progression. This field provides essential insights into how changes at the metabolic level can promote the proliferation and metastasis of cancer cells [4].

In LC, specific alterations in glycolysis and lipid metabolism have been identified as pivotal to the disease’s progression. These changes offer promising targets for therapeutic intervention due to their roles in supporting the heightened energetic and biosynthetic demands of cancer cells [5–7].Significant research, including work by Whitehouse et al., illustrates the potential of targeting key metabolites for cancer treatment. For instance, manipulating metabolites such as dichloroacetate (DCA), which affects the activity of pyruvate dehydrogenase, demonstrates a viable method to alter cancer cell metabolism effectively [8, 9]. However, the specific causal relationships between these metabolic changes and the risk of developing LC are still not well established, with only a few prospective studies linking metabolomic profiles directly to cancer progression. Advances in genome-wide association studies (GWAS) and the application of Mendelian randomization (MR) have provided new avenues for exploring the genetic foundations of metabolic changes in human diseases, including cancer [10, 11]. MR, in particular, offers a robust methodology for identifying potential causal relationships between metabolic disruptions and LC by using genetic variants as proxies to reduce confounding factors in observational studies [12].

Our research integrates MR with extensive GWAS data to assess how specific blood metabolites might influence the risk of LC, aiming to uncover the genetic and proteomic mechanisms that link these metabolic changes to the disease. This comprehensive approach allows us to explore the intricate network of genetic interactions and metabolic pathways, potentially identifying biomarkers for early detection and novel targets for therapeutic intervention. Through this comprehensive approach, we aim to refine the accuracy of LC screening and tailor treatment strategies, thereby improving the prognosis and quality of life for LC patients.

## Materials and methods

### Study design

**Fig. 1:**
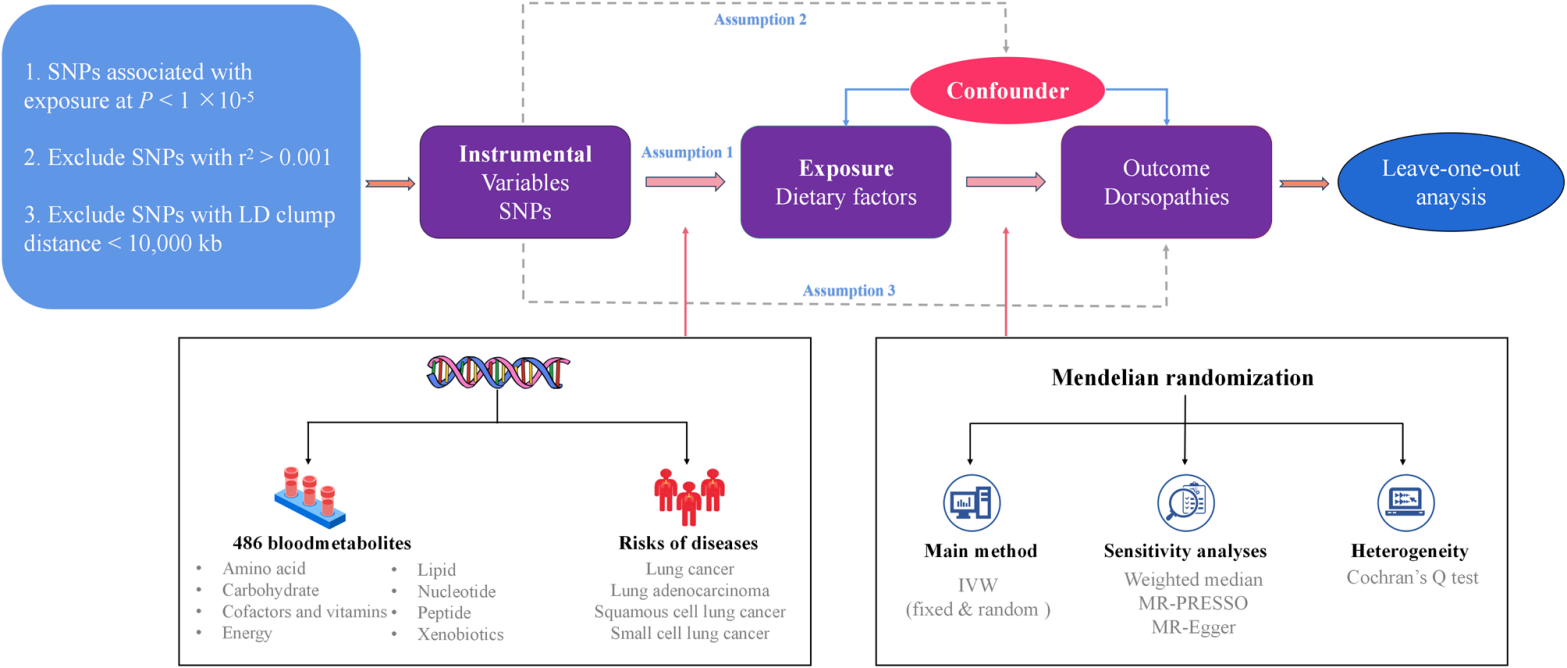
The overview of the research workflow

In our study, we selected single nucleotide polymorphisms (SNPs) from GWAS as genetic instrumental variables to investigate the potential causal relationship between human blood metabolites and LC [13]. The sequential workflow of our research is illustrated in Fig. 1. Our two-sample MR analysis is based on three main assumptions: (1) The assumption of relevance: IVs have a strong association with the exposure; (2) The assumption of independence: IVs are not correlated with any variables that may influence the exposure and outcome; (3) The assumption of exclusion restrictions: IVs do not affect the outcome through any other causal pathways, except for their effect on the exposure. All summary-level data used in our study are publicly accessible. Further ethical approval is not required as this study is based on publicly available GWAS data.

### Data source and study samples of lung cancer

This study’s findings are based on a GWAS conducted by the Transdisciplinary Research in Cancer of the Lung and the International Lung Cancer Consortium (TRICL-ILCCO) [14]. We included a total of 85,716 individuals, comprising 29,266 LC cases and 56,450 controls from TRICL-ILCCO. The study considered overall LC and its three major clinical subtypes: LC with 29,266 cases and 56,450 controls, lung adenocarcinoma (LUAD) with 11,273 cases and 55,483 controls, lung squamous cell cancer (LUSC) with 7,426 cases and 55,627 controls, and small cell lung cancer (SCLC) with 2,664 cases and 21,444 controls. To ensure the accuracy and reliability of the research results, this study rigorously checked and compared the participant IDs from the OncoArray dataset with those used in previous GWAS, including the ATBC, CARET, and Eagle studies. By this comparison, we ensured that the new dataset did not include any samples previously analyzed. This method prevents statistical biases and complexities that might arise from overlapping samples, thereby ensuring the accuracy and validity of the causal inferences derived from MR methods [14–16]. Additionally, we obtained summary data from the FinnGen consortium, which included 5,842 LC cases and 287,137 controls, analyzing genetic and health data from approximately 500,000 participants. The diagnosis of LC followed the International Classification of Diseases (ICD-10), as detailed in Supplementary Table S1. Our study primarily relied on publicly available GWAS summary data from populations of European descent. Through this analysis, we were able to confirm the key findings previously observed, which not only enhanced our confidence in the data analysis but also provided further evidence of the robustness and accuracy of our research conclusions.

### Genetic instruments selection

Genetic data on blood metabolites were obtained from the Metabolomics GWAS Server (https://metabolomics.helmholtz-muenchen.de/gwas/). This report represents the most comprehensive analysis of genetic loci for blood metabolites to date, identifying nearly 2.1 million SNPs across 486 metabolites, as determined by genome-wide association scans and high-throughput metabolic profiling by Shin et al. [17]. The names and chemical properties of these 486 metabolites, including those marked with “X-” whose properties are unknown, are listed in Supplementary Table S1. The study involved 7,824 participants of European ancestry, including 1,768 from the KORA F4 study in Germany and 6,056 from the UK Twin Study. Of the 486 metabolites, 107 are classified as unknown due to their poorly defined chemical properties [17]. According to the Kyoto Encyclopedia of Genes and Genomes (KEGG) database, the 309 identified metabolites are categorized into eight classes: cofactors and vitamins, energy, amino acids, carbohydrates, lipids, nucleotides, peptides, and xenobiotic metabolism [18]. Initial selection of instrumental variables (IVs) for each metabolite was based on a strict P-value cutoff of < 1 × 10^− 5^. SNPs with an r2 < 0.01 and a distance > 500 kb were retained after evaluating the linkage disequilibrium (LD). Additionally, the strength of the genetic instruments was evaluated using F-statistics to minimize bias from weak IVs. The F-statistic was calculated as (Beta/SE)^2^, with a mean value considered indicative of overall strength. An F-statistic > 10 denotes strong statistical power [19, 20]. Ultimately, 11,126 SNPs associated with the 486 blood metabolites were retained as IVs. Detailed information on the IVs for the 486 blood metabolites is provided in Supplementary Table S2.

### Statistical analyses

Four methods were used to assess the causal association between blood metabolites and four types of LC: inverse-variance-weighting (IVW), weighted median, maximum likelihood-based methods, and MR-Egger regression. The IVW approach, assuming all IVs are valid, combines their effects to produce an overall weighted effect [21]. Considering potential heterogeneity, both random and fixed effect models of IVW were calculated as main analyses. The weighted median estimator provides robust causal estimates even when up to 50% of the IVs are invalid [22]. Additionally, assuming a linear relationship between exposure and outcome, the maximum likelihood-based method estimates causal associations using a normal bivariate distribution [23]. The MR Egger method includes an intercept term in the regression model to evaluate directional pleiotropy [24]. An intercept significantly different from zero suggests pleiotropy and a violation of the basic MR assumption. Heterogeneity among IVs was assessed using Cochran’s Q test [25]. If substantial heterogeneity was found (*P* < 0.05), a random-effects model was used; otherwise, a fixed-effects model was employed (*P* > 0.05). Leave-one-out analysis identified influential SNPs affecting causal estimates, with a significance threshold set at *P* < 0.05 (two-sided). If fewer than four SNPs are available, only the IVW method is used. All analyses utilized the Mendelian Randomization, MRPRESSO, TwoSampleMR, and ggplot2 packages in R software (version 3.6.3).

## Results

### Causal estimates of genetically predicted blood metabolites on lung cancer

**Fig. 2:**
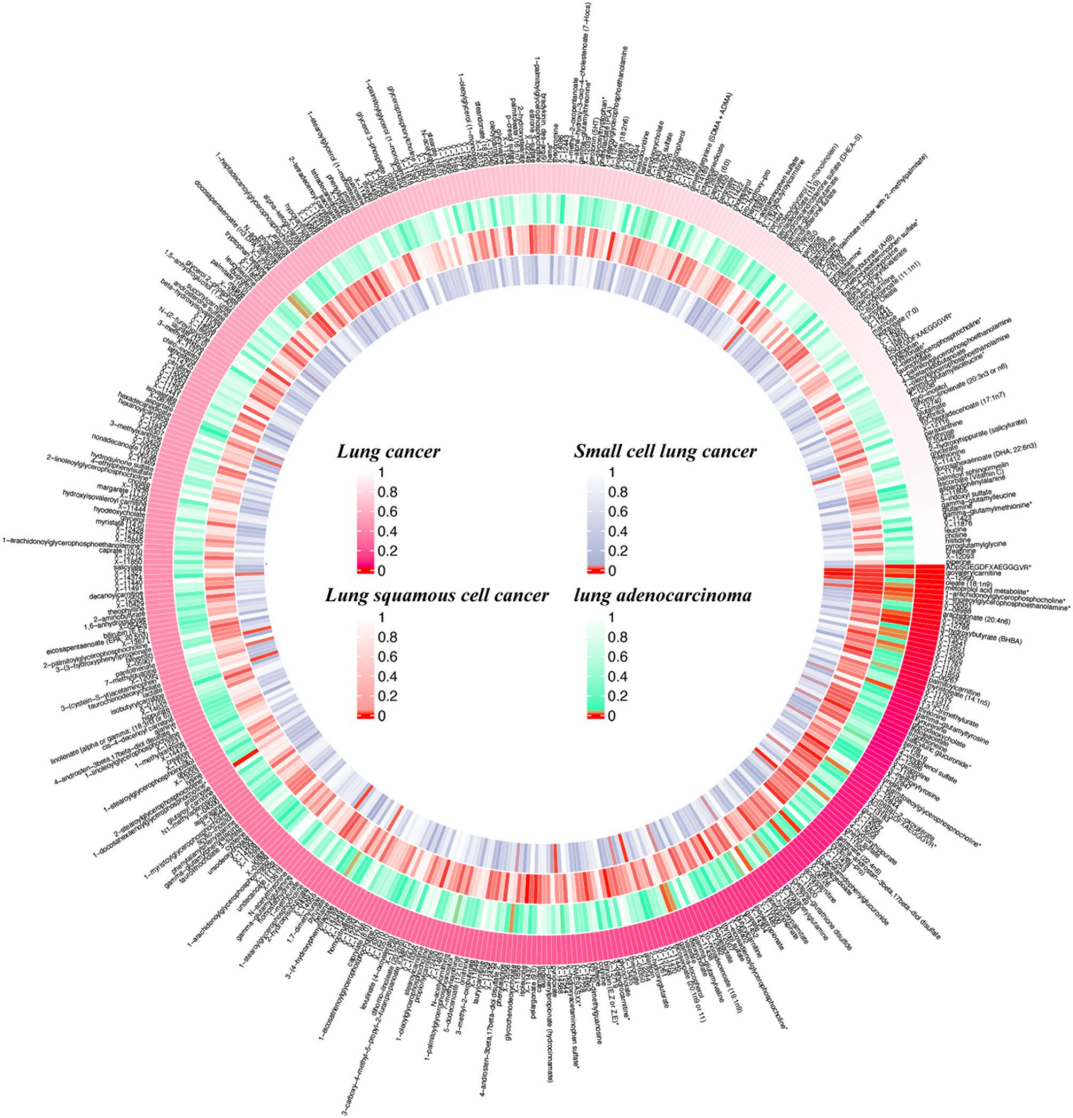
Circular Manhattan plot displaying the associations between blood metabolites and the risk of LC

Initially, to elucidate the association between metabolic alterations and LC, we employed the IVW method in our primary MR analysis. This method facilitated the estimation of causal relationships between 486 blood metabolites, including amino acids, carbohydrates, cofactors and vitamins, energy, lipids, nucleotides, peptides, and xenobiotic metabolism, and the risk associated with overall LC and its three specific subtypes: LUAD, LUSC, and SCLC. These analyses are illustrated in Fig. 2. Subsequently, to advance our understanding of LC prevention and diagnosis, we conducted a detailed analysis focused on how these 486 metabolites are associated with LC susceptibility overall and in these subtypes. This comprehensive approach aimed to pinpoint key biomarkers crucial for the early detection and prevention of LC. Understanding these associations is critical, as these biomarkers can significantly improve diagnostic processes and potentially provide guidance for preventive measures in clinical settings.

**Fig. 3:**
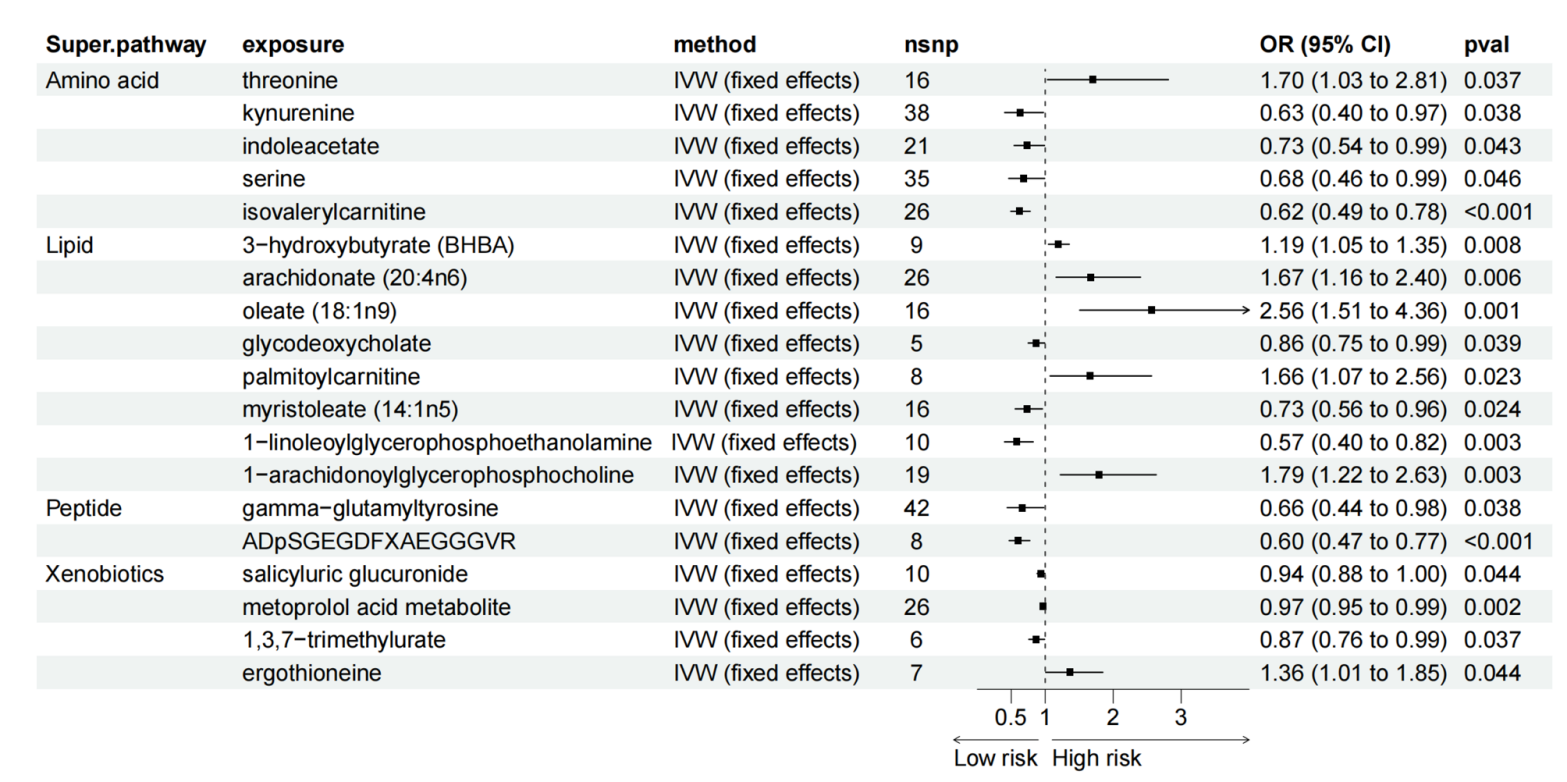
Forest plot for the causality of blood metabolites on LC derived from IVW analysis. CI, confidence interval; IVW, inverse variance weighted; OR, odds ratio; SNPs, single nucleotide polymorphisms

By identifying metabolites with the most significant associations, our research endeavors to provide actionable insights that could foster more effective strategies for managing LC risk and enhancing patient outcomes. As shown in Fig. 3, a total of 19 blood metabolites were identified with significant associations with LC risk using the IVW method. Specifically, genetically predicted levels of 1-linoleoylglycerophosphoethanolamine (OR = 0.57; 95% CI = 0.40–0.82, *P* = 0.003), ADpSGEGDFXAEGGGVR (a fibrinogen cleavage peptide, OR = 0.60; 95% CI = 0.47–0.77, *P* < 0.001), and isovalerylcarnitine (OR = 0.62; 95% CI = 0.49–0.78, *P* < 0.001) were associated with lower risk of LC. These associations were consistent across additional analytical methods such as Maximum Likelihood and Weighted Median, detailed in Supplementary Table S3. Conversely, higher risk were associated with the metabolites oleate (OR = 2.56; 95% CI = 1.51–4.36, *P* = 0.001), 1-arachidonoylglycerophosphocholine (OR = 1.79; 95% CI = 1.22–2.65, *P* = 0.003), and arachidonate (OR = 1.67; 95% CI = 1.16–2.40, *P* = 0.006). Similar findings were observed for the association of ergothioneine with LC risk. Heterogeneity and pleiotropy tests showed no significant influence on the causal effects of the metabolites on LC (*P* > 0.05). Additionally, the MR-PRESSO global test suggested no outlier SNPs, reinforcing the robustness of our findings.

### Causal estimates of genetically predicted blood metabolites on subtype lung cancer

**Fig. 4:**
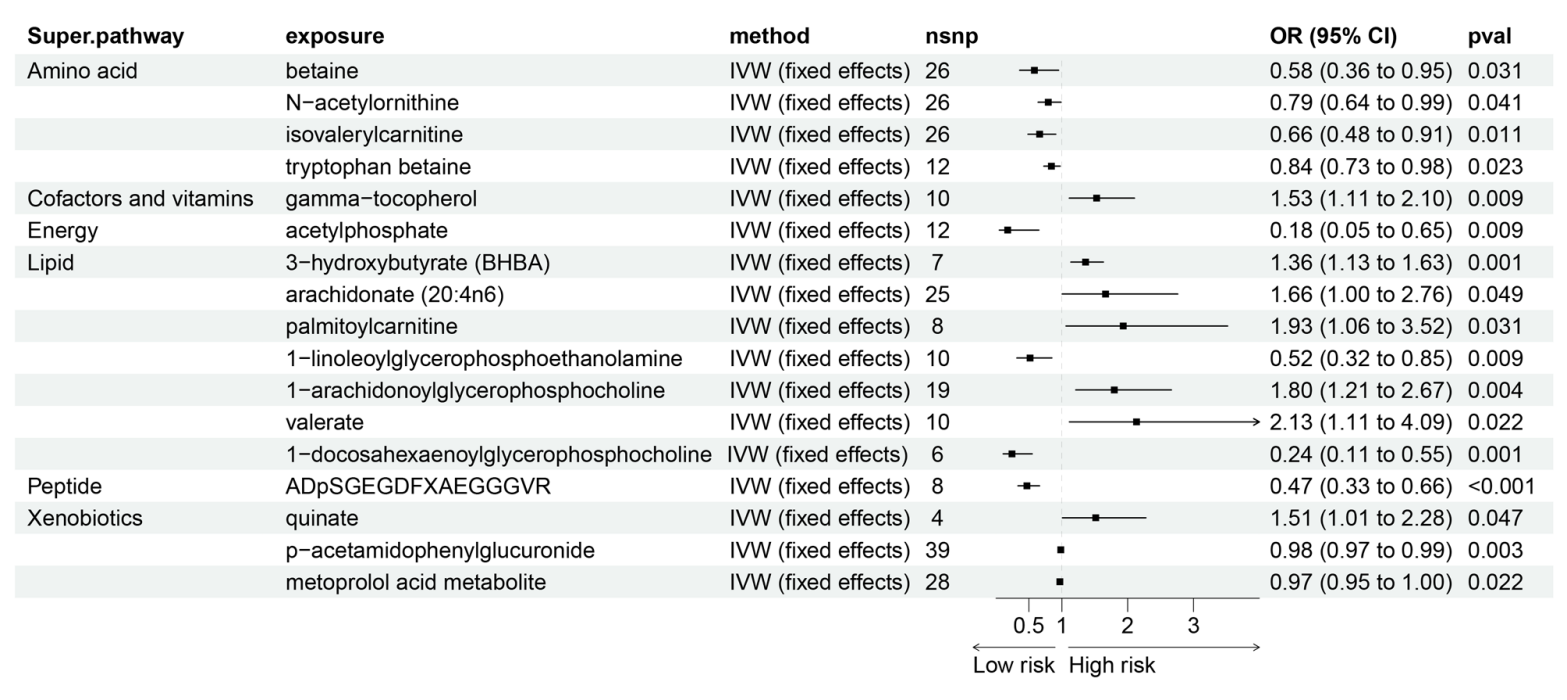
Forest plot for the causality of blood metabolites on LUAD derived from IVW analysis. CI, confidence interval; IVW, inverse variance weighted; OR, odds ratio; SNPs, single nucleotide polymorphisms

**Fig. 5:**
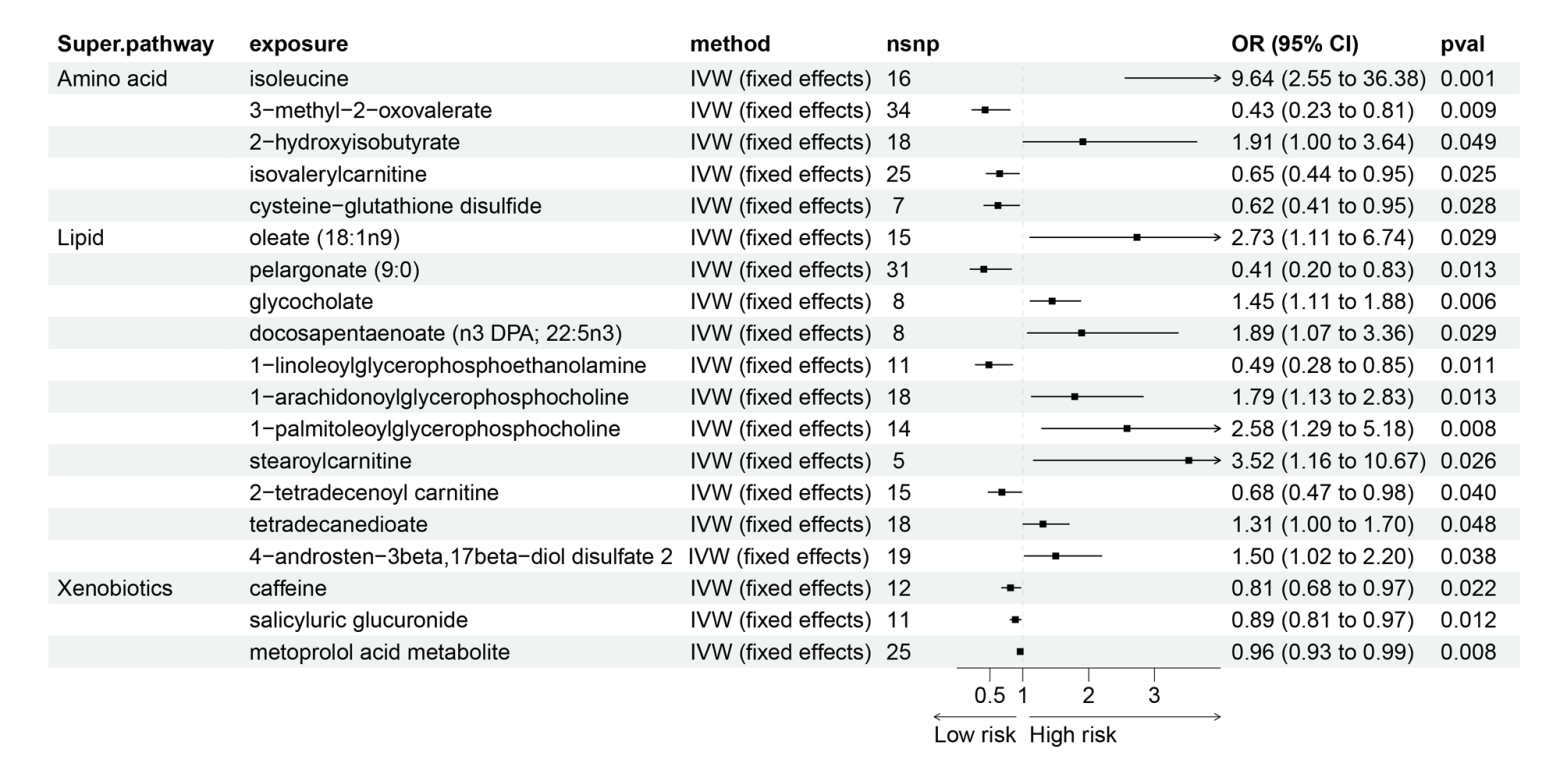
Forest plot for the causality of blood metabolites on LUSC derived from IVW analysis. CI, confidence interval; IVW, inverse variance weighted; OR, odds ratio; SNPs, single nucleotide polymorphisms

**Fig. 6:**
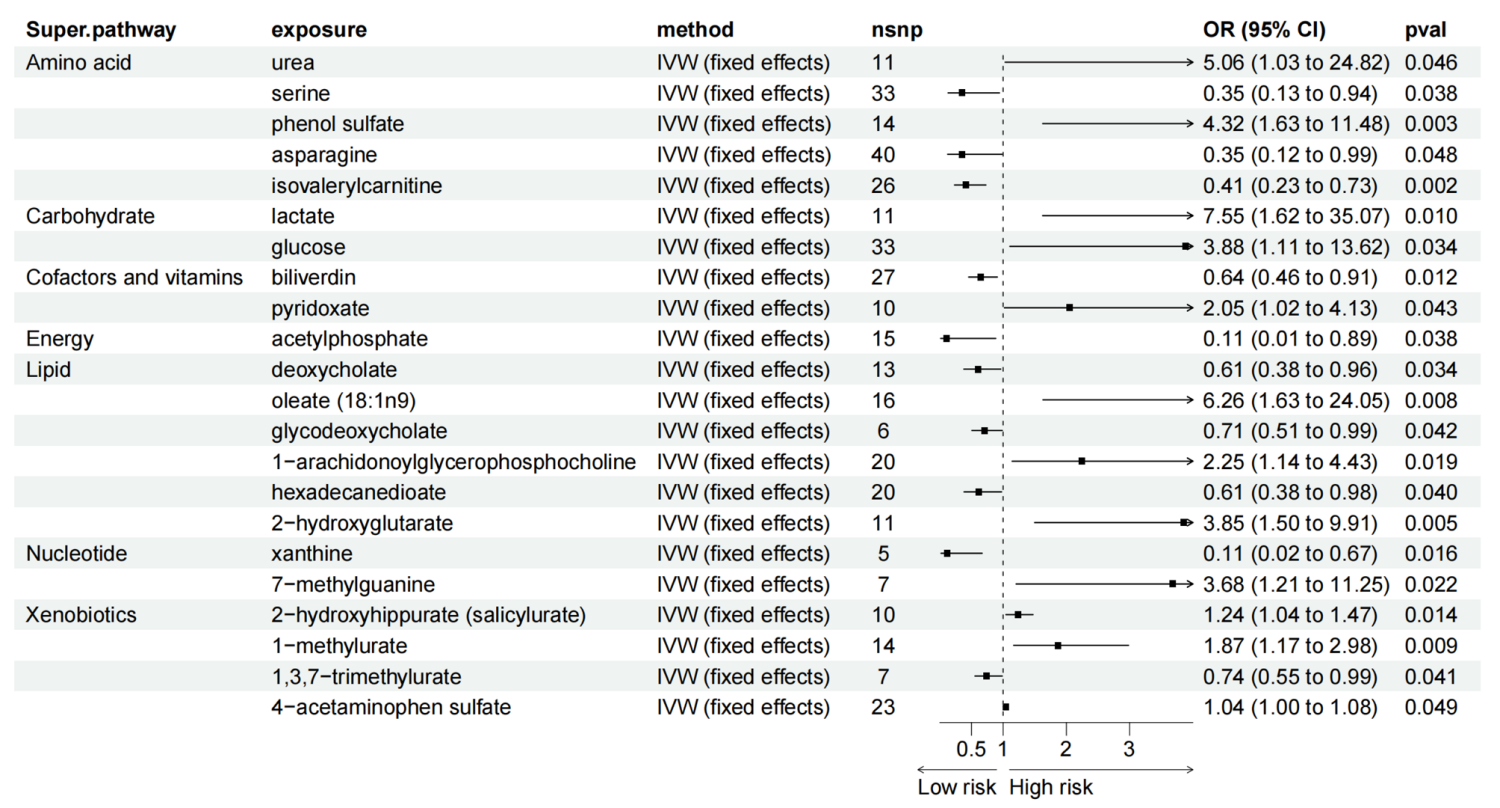
Forest plot for the causality of blood metabolites on SCLC derived from IVW analysis. CI, confidence interval; IVW, inverse variance weighted; OR, odds ratio; SNPs, single nucleotide polymorphisms

The influence of all blood metabolites on LC subtypes is detailed in Supplementary Tables S4–S6. Figures 4–6 provide visual representations from the MR analysis, indicating that 50 blood metabolites are linked to specific LC subtypes. Notably, 1-arachidonoylglyceropholine was associated with higher risk across multiple subtypes: SCLC with an OR of 2.25 (95% CI = 1.14–4.43, *P* = 0.019), LUSC with an OR of 1.79 (95% CI = 1.13–2.83, *P* = 0.013), and LUAD with an OR of 1.80 (95% CI = 1.21–2.67, *P* = 0.004). Conversely, isovalerylcarnitine was associated with lower risk for all three subtypes, showing protective associations as observed using the IVW method: LUAD (OR = 0.66, 95% CI = 0.48–0.91, P = 0.011), LUSC (OR = 0.65, 95% CI = 0.44–0.95, P = 0.025), and SCLC (OR = 0.41, 95% CI = 0.23–0.73, P = 0.002).

Additionally, 1-linoleoylglycerophosphoethanolamine was identified as a significant protective factor for LUAD (OR = 0.52, 95% CI = 0.32–0.85, P = 0.009) and LUSC (OR = 0.49, 95% CI = 0.28–0.85, P = 0.011). These associations were primarily confirmed through the Maximum Likelihood method (Supplementary Tables S4–S6). Notably, isoleucine (OR = 9.64, 95% CI: 2.55 to 36.38) was associated with a significantly higher risk of LUSC. For SCLC, the IVW method suggested that genetically predicted oleate was associated with a substantially higher risk (OR = 6.26, 95% CI = 1.63–24.05, *P* = 0.008), while acetylphosphate was associated with a significant reduction in risk (OR = 0.11, 95% CI = 0.01–0.89, *P* = 0.038). Similar associations were corroborated with MR-PRESSO and Maximum Likelihood analyses. Genetically predicted acetylphosphate also showed a strong protective association against LUAD, with IVW estimates indicating a significant reduction in risk (OR = 0.18, 95% CI = 0.05–0.65, *P* = 0.009; Fig. 4). Additionally, the metoprolol acid metabolite was associated with slightly lower risk for both LUSC (OR = 0.96, 95% CI = 0.93–0.99, *P* = 0.008; Fig. 5) and LUAD (OR = 0.97, 95% CI = 0.95-1.00, *P* = 0.022; (Fig. 4). The pleiotropy tests, including the Egger intercept analysis, indicated no significant influence on the associations (*P* > 0.05).

### Heterogeneity and sensitivity analysis

**Fig. 7:**
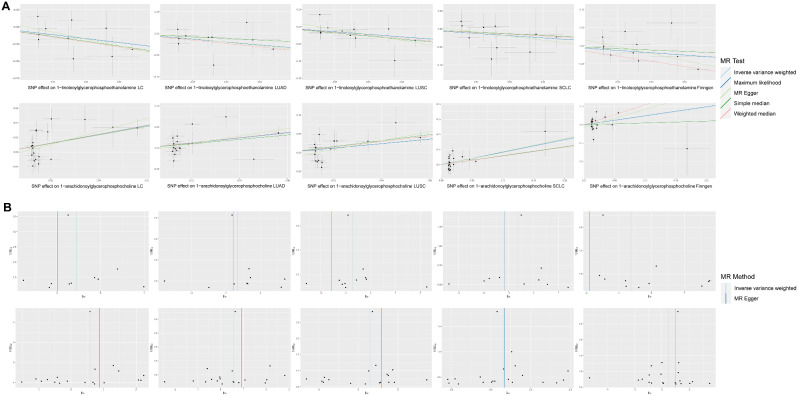
Genetic association of blood metabolites with overall LC and the risk of its three major clinical subtypes. (**A**) Scatter plot of overall LC and its three major clinical subtypes. (**B)** Funnel plot of overall LC and its three major clinical subtypes

While the IVW method is effectively used to estimate associations between exposures and disease outcomes, it is still vulnerable to biases arising from weak instrumental variables. To ensure the reliability of our conclusions, we employed data from the FinnGen consortium and conducted comprehensive sensitivity analyses to validate the principal findings from the TRICL-ILCCO studies. In the FinnGen dataset, we confirmed that 1-linoleoylglycerophosphoethanolamine is negatively associated with LC risk (OR = 0.59; 95% CI = 0.35–0.98, *P* = 0.042). Similarly, 1-arachidonoylglycerophosphocholine was found to be positively associated with LC risk (OR = 1.66; 95% CI = 1.12–2.47, *P* = 0.013), as illustrated in Supplemental Fig. S1. To further assess the robustness of these findings, we conducted extensive sensitivity and heterogeneity analyses across both datasets. As depicted in Fig. 7, scatter plots and funnel plots consistently demonstrated robust results, showing no significant influence from any single SNP. These findings not only reinforce the consistency and reliability of our observations but also highlight the statistical rigor applied throughout our research.

## Discussion

LC remains a major global health challenge, with an ever-growing need for effective screening and prevention strategies [1–3]. While risk factors such as smoking and environmental elements have been identified, the specific etiology of many cases remains elusive. Our study, employing MR and GWAS data, conducted an in-depth exploration of the causal relationship between blood metabolites and LC. This is the first systematic assessment of the role of human blood metabolites in the onset of LC, revealing how key metabolic disturbances potentially accelerate LC progression. Our findings expose the complex interplay between LC risk and metabolic changes, offering new insights into the metabolic pathology of LC. Our research identified 19 blood metabolites with significant associations with LC risk. Specifically, 1-linoleoyl glycerophosphoethanolamine, ADpSGEGDFXAEGGGVR (a fibrinogen cleavage peptide), and isovalerywere found to be associated with lower risks of LC, showing reductions in risk of 43%, 40%, and 38%, respectively. Conversely, oleate, 1-arachidonoylglyceropholine, and arachidonate were linked to increased LC risks, at 2.56, 1.79, and 1.67 times the original risk, respectively. Notably, isoleucine was associated with an increase to 9.64 times the original risk of LC. In the case of SCLC, genetically predicted oleate was found to increase the risk to 6.26 times the original. Acetylphosphate significantly lowered the risk, reducing it by 89%. 1-arachidonoylglyceropholine markedly elevated the risk across all LC subtypes, highlighting its potential as a biomarker in LC prevention and treatment strategies. Isovalerylcarnitine demonstrated a protective effect across all LC subtypes, underscoring its potential as a protective biomarker in LC prevention and treatment strategies.

Linoleoylglycerophosphoethanolamine, a key member of the phosphatidylethanolamine (PE) family, consists of fatty acids, ethanolamine, phosphate, and glycerol [26]. Its high expression in colorectal cancer tissues may be linked to cancer progression [27]. Research indicates that elevated serum levels of 1-linoleoylglycerophosphoethanolamine reduce the risk of atherosclerosis and renal failure [28]. As a fundamental component of cell membrane phospholipids, PE is essential for maintaining cellular structural stability. PE and its precursors, ethanolamine and ethanolamine phosphate, have demonstrated effectiveness in inhibiting the proliferation and metastasis of various cancer cells [29, 30]. Ethanolamine intervention in a colon xenograft mouse model significantly reduced tumor size. AdpSGEGDFXAEGGGVR has been associated with reduced risks of prostate and pancreatic cancers, establishing it as a potential blood metabolite for future testing [31–33]. Isovalerylcarnitine, synthesized in the human kidneys and liver, is vital for cellular energy metabolism. It facilitates the transport of long-chain fatty acids into mitochondria for β-oxidation and regulates the balance between free coenzyme A and acyl-coenzyme A [34, 35]. Abnormalities in acylcarnitine levels suggest disorders in fatty acid β-oxidation and branched-chain amino acid metabolism, linked to metabolic diseases such as leucine and isoleucine metabolism disorders, isovaleric acidemia, and type 2 diabetes [26–29]. Carnitine is proposed to stimulate neuroprotective factors [36]. Previous research has shown that isovalerylcarnitine activates the calpain system, significantly increasing early apoptosis and cytotoxicity [37]. 1-arachidonoylglycerophosphocholine, an important lyso-phosphatidylcholine (LPC), has been reported to inhibit CXCR3-mediated T cell migration to inflammatory sites [38]. In healthy individuals, plasma levels of LPC range from 125 to 143 nmole/mL, but these levels are elevated in conditions such as cardiovascular disease, diabetes, ovarian cancer, and renal failure [39–42]. Other studies suggest that higher levels of arachidonoylglycerophosphocholine are linked to thyroid and colon tumors. Oleate is known for its extensive physiological regulatory functions, stimulating fatty acid transport protein 1-mediated fatty acid uptake and inhibiting ATP-binding cassette transporters ABCA1/G1-mediated cholesterol efflux, leading to accumulation of neutral lipids, fatty acids, total cholesterol, and cholesterol esters in macrophages, thus influencing disease progression [43–45]. Our study’s findings echo recent research emphasizing the importance of metabolic reprogramming in LC progression. Recent studies suggest that targeting energy metabolism pathways in LC cells could be key therapeutic targets; our findings lend further support to these theories. Additionally, our results align with cancer metabolism research, indicating that metabolites could serve as potential biomarkers.

This study is innovative for several reasons. First, it uniquely combines metabolomics and genomics through Mendelian randomization to elucidate the causal relationships between blood metabolites and various types of LC, offering substantial clinical research value. Second, the employment of diverse MR models and stringent quality control measures enhances the reliability and stability of our findings. Despite these strengths, the study faces challenges due to the extensive variety of metabolites analyzed, complicating the data interpretation. Furthermore, while our results provide foundational insights, there remains a critical need for further research to elucidate the specific biological mechanisms through which these metabolites influence LC progression, including their roles in cell proliferation, invasion, and resistance. Future studies should also examine these metabolites across different populations and cancer subtypes to fully understand their impacts and potential clinical applications.

## Conclusion

Our study provides a novel understanding of the metabolic underpinnings of LC by demonstrating a potential causal link between specific blood metabolites and the risk of developing LC. These insights are pivotal for advancing early detection and preventative strategies. Additionally, our findings could significantly inform the development of targeted treatment approaches, potentially leading to more personalized and effective therapeutic options for LC patients.

### Electronic supplementary material

Below is the link to the electronic supplementary material.

Supplementary Material 1

Supplementary Material 2

## Data Availability

All data generated or analyzed during this study are included in this published article and its supplementary information files.
