# Supplementary figures and images for: The associations between dysregulation of human blood metabolites and lung cancer risk: evidence from genetic data
Source: BMC Cancer. 2024 Jul 18;24:854. doi: 10.1186/s12885-024-12416-1 (PMC11256634; doi:10.1186/s12885-024-12416-1)

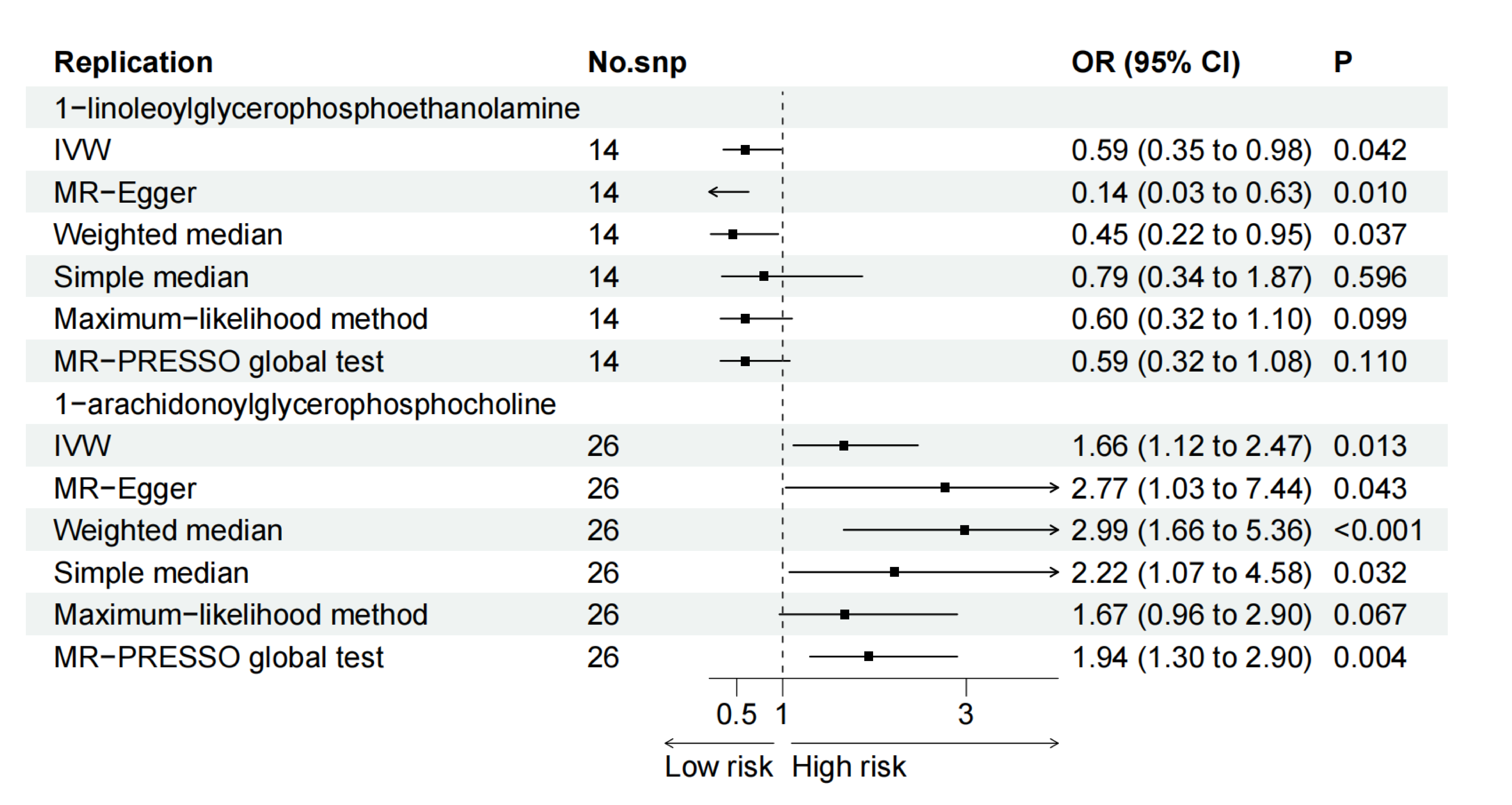

Supplement: Supplementary file 1 — Supplementary Material 1 [file 12885_2024_12416_MOESM1_ESM.png]
